# ITGAL as a prognostic biomarker correlated with immune infiltrates in melanoma

**DOI:** 10.3389/fonc.2023.1181537

**Published:** 2023-06-14

**Authors:** TengFei Deng, Chaoyong Wang, Cong Gao, Qiang Zhang, Jun Guo

**Affiliations:** ^1^ Plastic Surgery Department, Yangzhou University Affiliated Hospital, Yangzhou, China; ^2^ Medical College of Yangzhou University, Yangzhou, China

**Keywords:** ITGAL, melanoma, prognostic biomarker, immune infiltrates, MIHC

## Abstract

This study investigates the relationship between ITGAL expression and immune infiltration, clinical prognosis, and specific types of T cells in melanoma tissue. The findings reveal the key role of ITGAL in melanoma and its potential mechanism of regulating tumor immune infiltrating cells, highlighting its potential as a diagnostic biomarker and therapeutic target for advanced melanoma.

## 1 Background

Malignant melanoma is a common, highly malignant tumor of the skin and mucosa, and its incidence rate ranks third among skin tumors (1). At present, the number of patients increases by 3%–5% every year, making it the fastest-growing malignant tumor in the world (2, 3). The 5-year overall survival rate of patients with melanoma is 92%, and the 5-year survival rate of patients with advanced metastasis (stage IV) is 23% (4). With the emergence of new treatment methods and targeted drugs, it has brought new hope and a breakthrough to treatment (5, 6). However, because malignant melanoma is prone to recurrence and metastasis, any specific treatment cannot improve the overall survival rate (7). To solve the difficulties in the treatment of melanoma, it is necessary to explore potential biomarkers related to the prognosis of melanoma patients and serve as potential therapeutic targets for these patients.

ITGAL, also known as CD11a, encodes an integrin component of lymphocyte function-associated antigen-1 (LFA-1). ITGAL was highly expressed on the surface of activated CD4+T cells in peripheral blood, damaged skin, and gastric mucosa (8, 9), regulating intercellular adhesion and lymphocyte costimulatory signals (10, 11). Some studies have proved that ITGAL is related to the occurrence and progression of tumors (12, 13), including accelerating the cell cycle process (13), participating in immune reactions, and affecting the tumor microenvironment (14–16). It has the potential to become a new target for malignant tumors. However, the pathogenesis of ITGAL and melanoma is still unclear.

In this study, we combined various databases and biological experiments to study the relationship between the expression of ITGAL and immune infiltration in melanoma, and performed functional analysis of ITGAL-mediated TCGA-SKCM data. In addition, the relationship between the expression of ITGAL and CD4+ CD8+T cells in melanoma tissue sections was studied by multiple immunofluorescence staining. This study revealed the key role of ITGAL in melanoma and the mechanism by which ITGAL may regulate tumor immune infiltrating cells.

## 2 Materials and methods

### 2.1 Data acquisition

GTEx gene expression data, TCGA-SKCM (FPKM) gene expression data, and related clinical characteristics data were downloaded from the UCSC Xena database (https://xenabrowser.net/datapages/). Patients with incomplete clinical data were not included. We obtained the data on log2 (x + 0.001) conversion. A total of 472 patients (all from TCGA) and 556 normal controls (all from GTEx data, including non-sun-exposed suprapubic skin and sun-exposed lower leg skin) were included in this study. The gene names of all expression matrix data were annotated through the “ClusterProfiler” and “org.Hs.eg.db” software packages (17).

### 2.2 Material preparation

Melanoma B16 cell line and mouse fibroblast NIH/3T3 cell line were obtained from Beyotime (https://www.beyotime.com) and stored in RPMI-1640 (Gibco) containing 10% fetal bovine serum and 1% penicillin–streptomycin.

The 98 cases of fresh tissue after surgery were collected from the Affiliated Hospital of Yangzhou University, including 90 cases of melanoma tissue and eight cases of normal tissue. No patient received radiotherapy or chemotherapy before tissue collection. Each tissue from six samples was divided into two parts, one for WB and the rest to produce the tissue microarray (TMA).

### 2.3 GEPIA online database

Gene Expression Profiling Interactive Analysis (GEPIA) (http://gepia.cancer-pku.cn) is a public database developed by Peking University to compare gene expression differences between normal and tumor tissues. The dataset includes RNA sequencing and expression data from more than 9,000 tumor samples and 8,000 normal samples from TCGA and GTEx, including 33 malignant tumors (18). This study used the GEPIA database to analyze the expression level of ITGAL in TCGA-SKCM and its relationship to the prognosis and survival of patients.

### 2.4 Tumor immune estimation resource database analysis

*The Tumor Immune Estimation Resource (TIMER2.0)* database can explore the molecular characterization of different immune cells in various cancer types and analyze the correlation between gene expression and the infiltration of various types of immune cells, including B cells, CD8+T cells, CD4+T cells, monocytes, neutrophils, and dendritic cells (19).

### 2.5 TNMplot online database

The TNMplot online database (https://www.tnmplot.com) is a web tool for the comparison of gene expression in normal, tumor, and metastatic tissues. It uses multiple RNA-seq and microarray datasets to establish the largest transcriptomic cancer database at present, including nearly 57,000 samples, and it can explore any gene database to evaluate the expression differences in normal, cancer, and metastatic samples (20).

### 2.6 GSEA analysis for KEGG and GO functional analysis

*Gene Set Enrichment Analysis (GSEA)* is a computational method that determines whether *a priori*-defined set of genes shows statistically significant and concordant differences between two biological states. Gene set enrichment analysis is a method to infer biological pathway activity from gene expression data (21). According to the expression of ITGAL in the sample data, we divide the TCGA-SKCM dataset into ITGAL-HIGH and ITGAL-LOW groups, then Limma R package (22) for differential gene (DEG) analysis, R package ClusterProfiler (17, 23) for GSEA enrichment analysis, including Gene Ontology (GO) and Kyoto Encyclopedia of Genes and Genomes (KEGG) path analysis for DEGs. We specified an adjusted P-value of <0.05 and an FDR q-value of <0.25 to be statistically significant.

### 2.7 Protein–protein interaction network analysis

We use the STRING (https://stringdb.org/) (24) online tool to select multiple proteins in *Homo sapiens* to analyze the protein–protein interaction (PPI) network of differentially expressed mRNA target genes. Cytoscape 3.8 and the cytoHubba plug-in are used for PPI network topology analysis, and finally, the top 10 hub target genes with high connectivity are selected.

### 2.8 Western blotting

Protease inhibitors were used in three cases of melanoma tissue and three cases of normal tissue in the radioimmunoprecipitation assay buffer. Prepare a 10% SDS-PAGE gel containing equal amounts of protein (30 µg protein from cell lysate or 40 ng purified protein), transfer it to a PVDF membrane after electrophoresis, and add 5% BSA for blocking buffer. Then add rabbit anti-ITGAL (1∶1,000) and GAPDH (1∶2,000) and put them in a refrigerator at 4 °C overnight. The following antibodies were used: rabbit anti-ITGAL (EP1285Y, Abcam) and rabbit anti-GAPDH (ab245355, Abcam).

### 2.9 Real‐time quantitative polymerase chain reaction

We used TRIzol reagent (Invitrogen, USA) to extract total RNA from mouse melanoma B16 cell lines and mouse fibroblast NIH/3T3 cell lines for quantitative PCR (qRT-PCR). The primers used for qRT-PCR had reaction conditions of 95 °C for 30 s, 95 °C for 5 s, and 60 °C for 30 s, for a total of 40 cycles. The ITGAL primer sequence is as follows:

forward 5′-CTGCTTTGCCAGCCTCTCTGT-3′
reverse 5′-GCTCACAGGTATCTGGCTATGG-3′

The β-actin primer sequence is as follows:

forward 5’-GGCTGTATTCCCCTCCATCG-3’-3′
reverse 5’-CCAGTTGGTAACAATGCCATGT-3

using 2^−ΔΔ^ Ct method.

### 2.10 Multiplexed immunohistochemistry

The melanoma tissue microarray (TMA) (90 cases of melanoma tissues, eight cases of normal tissues) is used for mIHC staining. Firstly, the tissue section is used for dewaxing and repairing antigen, and fixed with an appropriate fixing solution, and then added with an immune staining blocking solution and antibody for MIHC staining. After triple fluorescence staining with red fluorescence, green fluorescence, and blue fluorescence, the slide was scanned with the Vectra 3.0 automatic quantitative pathological imaging system to detect and measure the positive rate of biomarkers: rabbit anti-ITGAL (EP1285Y, Abcam), rabbit anti-CD4 (EPR6855, Abcam), and rabbit anti-CD8 (CAL66, Abcam). The secondary antibody was Opal™ polymer HRP Ms+Rb (ARH1001EA, Perkin Elmer). Fluoroshield with DAPI (ab104139, Abcam) was used to stain the nuclei and seal the slices.

### 2.11 Statistical methods

In this study, the mean ± standard deviation (x ± s) was used to describe the expression of the ITGAL gene. The difference between the groups was analyzed by an independent t-test. The Kaplan–Meier method was used to determine the relationship between the expression of ITGAL and the prognosis of melanoma. A log-rank test was used for comparison between the groups. The difference between the two sides was statistically significant (P <0.05). Pearson correlation is used to evaluate the correlation between two gene expression data sets, and its value lies in the range of −1 to 1.

## 3 Results

### 3.1 Assessment of ITGAL expression in different cancer and normal tissues

**Figure 1:**
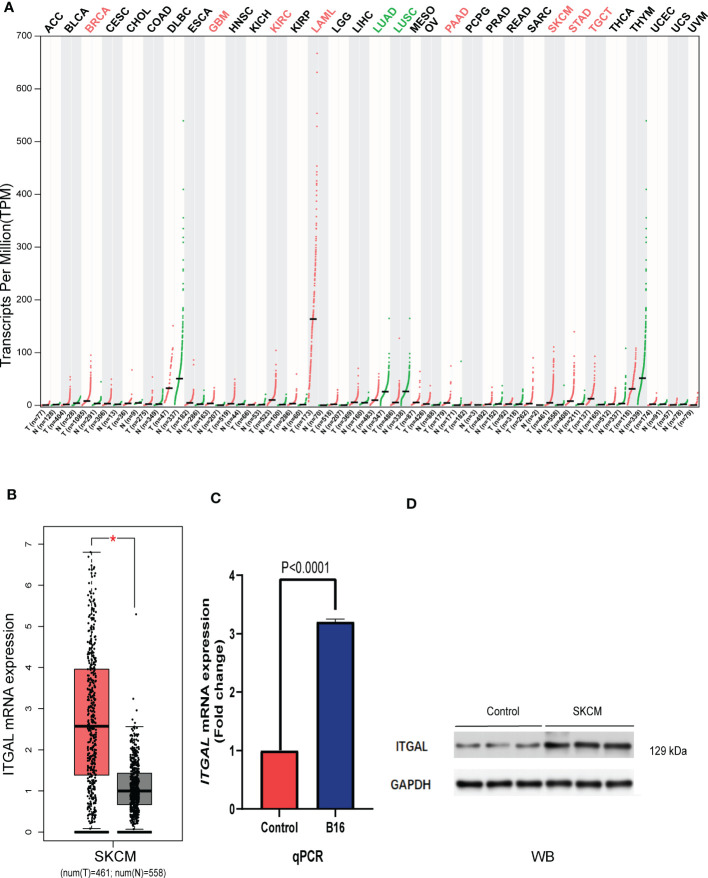
**(A)** ITGAL mRNA expression in pan-cancer. **(B)** ITGAL mRNA expression in TCGA-SKCM. **(C)** The qPCR result for the TGAL gene showed high expression in melanoma B16 cells. **(D)** The WB result for the TGAL gene showed high expression in melanoma tissue.

We first assessed the expression of the ITGAL gene in different tumors using the GEPIA database and found that the gene was lowly expressed in kidney chromophobe (KICH), lymphoid neoplasm diffuse large B-cell lymphoma (DLBC), lung adenocarcinoma (LUAD), lung squamous cell carcinoma (LUSC), rectum adenocarcinoma (READ), thyroid carcinoma (THCA), and thymoma (THYM) compared to normal tissues. We also found that ITGAL was highly expressed in bladder urothelial carcinoma (BLCA), breast invasive carcinoma (BRCA), cervical squamous cell carcinoma and endocervical adenocarcinoma (CESC), glioblastoma multiforme (GBM), head and neck squamous cell carcinoma (HNSC), kidney renal clear cell carcinoma (KIRC), kidney renal papillary cell carcinoma (KIRP), acute myeloid leukemia (LAML), brain lower grade glioma (LGG), liver hepatocellular carcinoma (LIHC), ovarian serous cystadenocarcinoma (OV), pancreatic adenocarcinoma (PAAD), sarcoma (SARC), skin cutaneous melanoma (SKCM), stomach adenocarcinoma (STAD), and testicular germ cell tumor (TGCT) compared to normal tissue controls (e.g., **Figure 1A**). We used the GEPIA database to validate the expression of ITGAL in melanoma (e.g., **Figure 1B**). Then the results of qPCR verified that ITGAL was highly expressed in B16 cells of melanoma (e.g., **Figure 1C**). Further, the results of Western blotting showed that ITGAL was highly expressed in melanoma compared to normal tissue controls (e.g., **Figure 1D**). These results suggest that ITGAL is highly expressed in melanoma tissues and may be a potential diagnostic biomarker for melanoma.

### 3.2 High expression of ITGAL is associated with clinicopathological features

Although histological classification or clinical stage can help predict the prognosis of melanoma patients, there is still a need to explore the relationship between ITGAL expression and clinical features.

**Figure 2:**
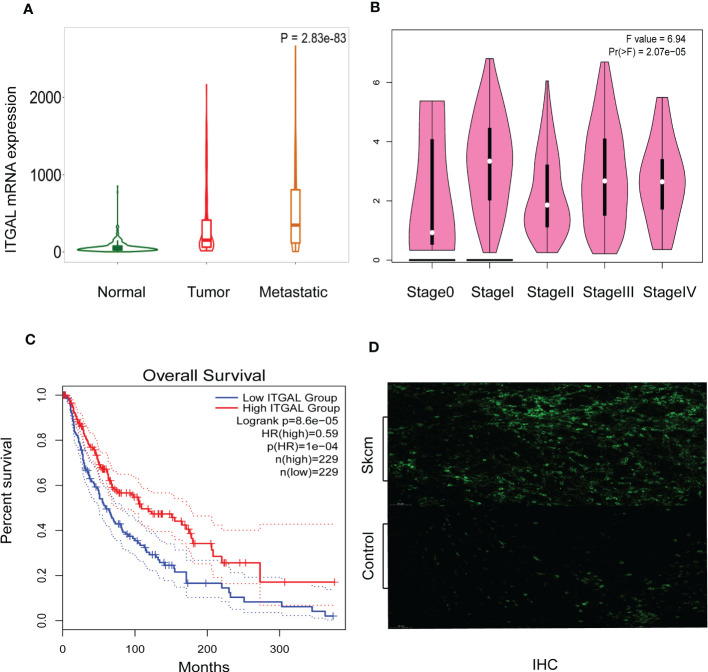
**(A)** ITGAL mRNA expression in normal, tumor, and metastatic tissues. **(B)** ITGAL mRNA expression in different stages of melanoma. **(C)** The Kaplan–Meier curve shows that the high ITGAL group had a better prognosis. **(D)** The IHC result for the TGAL gene showed high expression in melanoma tissue.

As shown in **Figure 2A**, TNMplotter database analysis showed that ITGAL gene expression increased sequentially in normal tissue, primary melanoma, and metastatic melanoma, which we further validated using immunohistochemical staining to obtain similar results (e.g., **Figure 2D**). Next, we analyzed the expression of ITGAL in different stages using the TCGA dataset of GEPIA. The results showed that ITGAL was significantly underexpressed in Stage II (e.g., **Figure 2B**). The results of **Figure 2C** indicate that high expression of ITGAL is associated with a good prognosis. This can be explained by the fact that high expression of ITGAL enhances the immune response.

We also analyzed the correlation between ITGAL expression and clinical–pathological features using clinical data from TCGA-SKCM after removing incomplete data. Detailed results are shown in **Table 1**.

**Table 1:** The correlation between ITGAL and clinicopathological features.

Characteristic	Total No.	Low or No Expression, No(%)	High Expression, No(%)	Pearson χ2	P-Value
Total No.	472	236(50.00)	236(50.00)		
Sex	472			2.158	0.14
Man	293	153	140		
Fenale	179	81	98		
Age(year)	471			4.671	***
≤60	249	112	137		
>60	222	122	100		
T	395			21.996	***
Ts+T1	72	21	51		
T2	79	39	40		
T3	91	42	49		
T4	153	95	58		
N	414			1.552	0.67
N0	235	122	113		
N1	74	38	36		
N2	49	22	27		
N3	56	25	31		
M	443			0.396	0.53
M0	418	207	211		
M1	25	14	11		
breslow_depth (mm)	360			21.61	***
< 1.0mm	50	12	38		
≥1mm and ≤4mm	167	79	88		
>4.0mm	143	88	55		
Clark's Level	322			17.239	***
Clark's Level I	6	3	3		
Clark's Level II:	18	3	15		
Clark's Level III	77	30	47		
Clark's Level IV	168	98	70		
Clark's Level V	53	30	23		

*** mean p<0.001.

These results suggest that ITGAL may be involved in the development of melanoma.

### 3.3 ITGAL expression correlated with immune cell infiltration in melanoma

**Figure 3:**
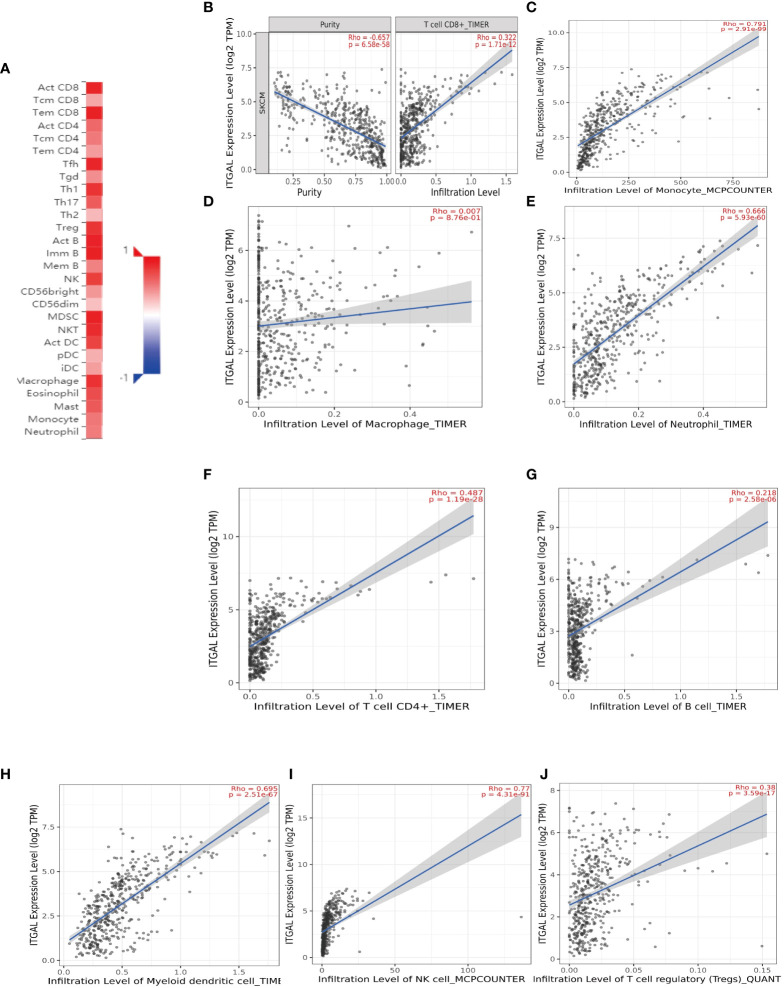
**(A)** The correlation between ITGAL mRNA expression and different TILs. **(B–J)** The correlation between ITGAL mRNA expression and different biomarkers of TILs.

The survival rate and lymph node metastasis rate are related to immune infiltration in melanoma (25–27). Therefore, we use the TIMER and TISIDB databases to explore the relationship between the expression of ITGAL and the immune infiltration in melanoma. In this study, the expression of ITGAL was negatively correlated with the purity of melanoma. The results showed that ITGAL was significantly correlated with most immune cells (see **Figure 3A**).

Among them, CD8+T cells (rho = 0.322), CD4+T cells (rho = 0.487), B cells (rho = 0.218), monocytes (rho = 0.791), neutrophils (rho = 0.666), T-cell regulation (rho = 0.38), NK cells (rho = 0.77), and myeloid dendritic cells (rho = 0.695) had a significant association with ITAGL. These results indicate that ITGAL plays an important role in the immune infiltration of melanoma. At the same time, we used the TIMER and GEPIA databases to explore the correlation between ITGAL and biomarkers of different tumor-infiltrating lymphocytes (TILs) in melanoma (e.g., **Figures 3B-L**). The results showed that ITGAL had a strong correlation with biomarkers of most immune cells. Further, we used GEPIA to verify and get similar results. We analyzed immune T cells (Th1/Th2/Th17/Tfh cells, Tregs, and exhausted T cells), as shown in **Table 2**.

**Table 2:** The correlation between ITGAL and biomarkers of immune cells.

Description	Gene marker	SKCM		SKIN(not sun exposed)	
		Cor	P	Cor	P
CD8+ T cell	CD8A	0.82	***	0.56	***
	CD8B	0.84	***	0.42	***
T cell (general)	CD3D	0.93	***	0.57	***
	CD3E	0.95	***	0.62	***
	CD2	0.93	***	0.73	***
B cell	CD19	0.6	***	0.11	0.087
	CD79A	0.64	***	0.35	***
Monocyte	CD86	0.79	***	0.47	***
	CD115(CSF1R)	0.6	***	0.63	***
TAM	CCL2	0.37	***	0.22	***
	CD68	0.39	***	0.32	***
	IL10	0.31	***	0.31	***
M1 Macrophage	CD80	0.72	***	0.43	***
	IRF5	0.65	***	0.23	***
	CD64	0.67	***	0.26	***
M2 Macrophage	CD163	0.45	***	0.25	***
	CD206	0.39	***	0.35	***
	VSIG4	0.49	***	0.29	***
	MS4A4A	0.57	***	0.42	***
Neutrophils	CD14	0.55	***	0.42	***
	CD11b(ITGAM)	0.57	***	0.48	***
	CCR7	0.66	***	0.15	***
Natural killer cell	IL2RB (CD122)	0.9	***	0.19	***
	CD244	0.57	***	0.44	***
	NCR1	0.41	***	0.43	***
	KLRD1	0.68	***	0.29	***
	ITGB2	0.84	***	0.47	***
Dendritic cell	CD209	0.53	***	0.49	***
	BTLA	0.6	***	0.53	***
	CLEC10A	0.71	***	0.71	***
	CD1C	0.57	***	0.62	***
	ID2	0.28	***	0.18	***
	CD11c(ITGAX)	0.58	***	0.44	***
Th1	T-bet(TBX21)	0.85	***	0.56	***
	STAT4	0.86	***	0.61	***
	STAT1	0.53	***	0.31	***
	IFN-r(IFNG)	0.72	***	0.28	***
	TNF-a(TNF)	0.54	***	0.06	***
Th2	GATA3	0.21	***	0.12	***
	STAT5A	0.27	***	0.27	***
	CCR3	0.67	***	0.11	***
	IL13	0.26	***	0.13	***
Tfh	BCL6	0.17	***	0.11	***
	IL21	0.7	***	0.04	***
Th17	BATF	0.6	***	0.28	***
	STAT3	0.16	***	0.15	***
Treg	FOXP3	0.77	***	0.29	***
	CCR8	0.66	***	0.34	***
	STAT5B	0.33	***	0.35	***
	TGFB(TGFB1)	0.36	***	0.2	***
T cell exhaustion	PD-1(PDCD1)	0.85	***	0.36	***
	PDL1(PDCD1LG2)	0.46	***	0.43	***
	CTLA4	0.2	***	0.39	***
	LAG3	0.72	***	0.22	***
	TIM-3(HAVCRP2)	0.78	***	0.48	***
	GZMB	0.57	***	0.5	***

*** mean p<0.001.

### 3.4 Results of mIHC confirmed that the expression status of ITGAL is correlated with the levels of CD8+ T cells and CD4+T cells

**Figure 4:**
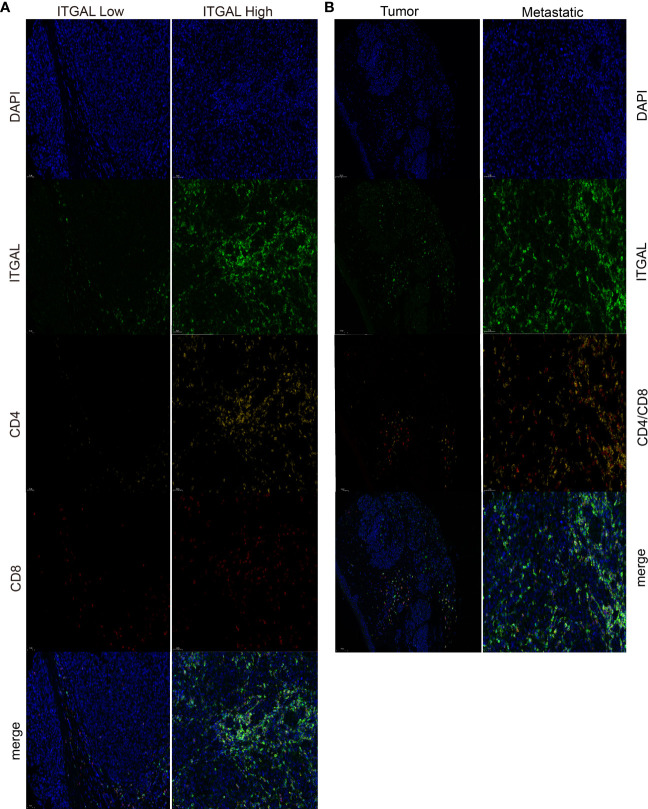
**(A)** The infiltration of CD4+T cells and CD8+T cells was significantly enhanced in the high-ITGAL group. **(B)** The infiltration of CD4+T cells and CD8+T cells was significantly enhanced in the metastatic melanoma group.

Next, we use mIHC technology to explore the relationship between ITAGAL and CD4+ and CD8+T cells in immune infiltrates in melanoma. We used computer image techniques to perform spatial analysis of different T cells at the same location of the tissue sample, and the results are shown in **Figure 4A**.

The results confirmed that almost all samples had different grades of immune cell infiltration. We divided samples into ITGAL-high and ITGAL-low expression groups according to the level of median expression of ITGAL. Compared with the low expression group of ITGAL, the infiltration of CD4+T cells and CD8+T cells in the high expression group of ITGAL was significantly enhanced (P <0.05).

In addition, we divided the sample into two groups: metastatic melanoma and primary melanoma. According to the expression of ITGAL in different groups, the results revealed that the level of immune cell infiltration increased with the progression of melanoma (e.g., **Figure 4**).

### 3.5 Relationships of ITGAL with immune checkpoint genes and cytokines

**Figure 5:**
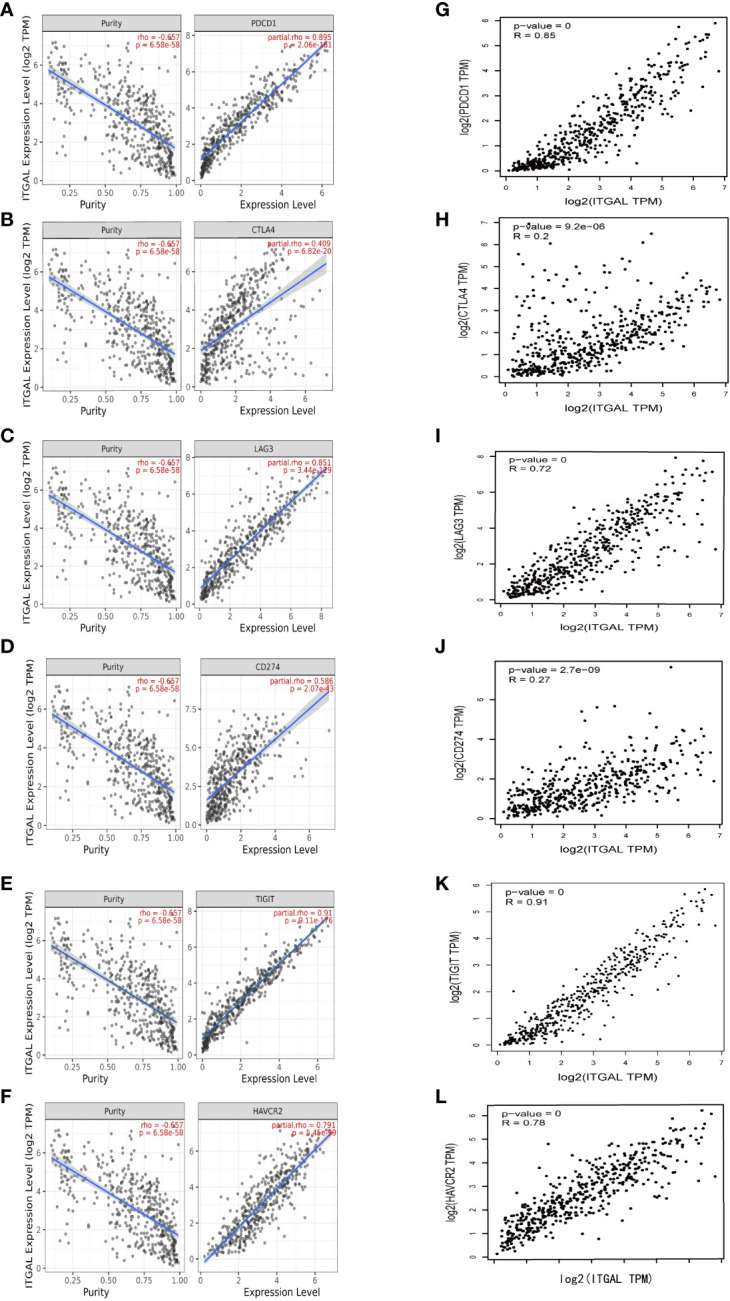
**(A–F)** The correlation between ITGAL and PD1, PD-L1, CTLA-4, LAG-3, TIM-3, and TIGIT in the TIMER2.0 database. **(G–L)** The correlation between ITGAL and PD1, PD-L1, CTLA-4, LAG-3, TIM-3, and TIGIT in the GEPIA2 database.

In addition to PD1/PD-L1, CTLA-4, LAG-3, TIM-3, and TIGIT are potential melanoma checkpoints in the future (28, 29). We used TIMER and GEPIA to explore the correlation between ITGAL and these immune checkpoints. As shown in **Figures 5A–F**, the expression level of ITGAL has a significant positive correlation with PD1/PD-L1, CTLA-4, LAG-3, TIM-3, and TIGIT. Then we used GEPIA to verify and get similar results (e.g., **Figures 5G-N**). These results suggest that tumor immune escape may participate in ITGAL-mediated melanoma.

### 3.6 Functional enrichment analysis of DEGs from high-ITGAL and low-ITGAL groups

**Figure 6:**
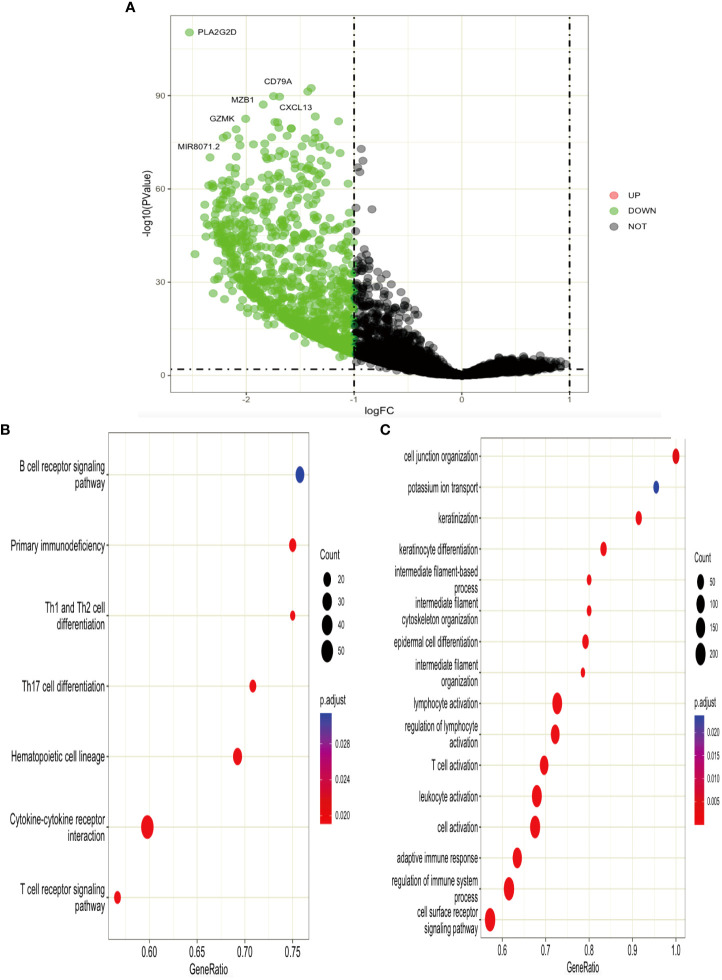
**(A)** Volcano plot of DEGs. **(B, C)** KEGG and GO functional analysis for DEGs.

According to the expression level of TGAL, we divided the TCGA-SKCM dataset into high-ITGAL and low-ITGAL groups. R packages limma for differential expression (DE) analysis; there are 1,000 DEGs in total, including two upregulated genes and 998 downregulated genes (e.g., **Figure 6A**). Then we perform KEGG and GO functional analysis, and the results are shown in **Figures 6B, C**.

### 3.7 Protein–protein interaction analysis

**Figure 7:**
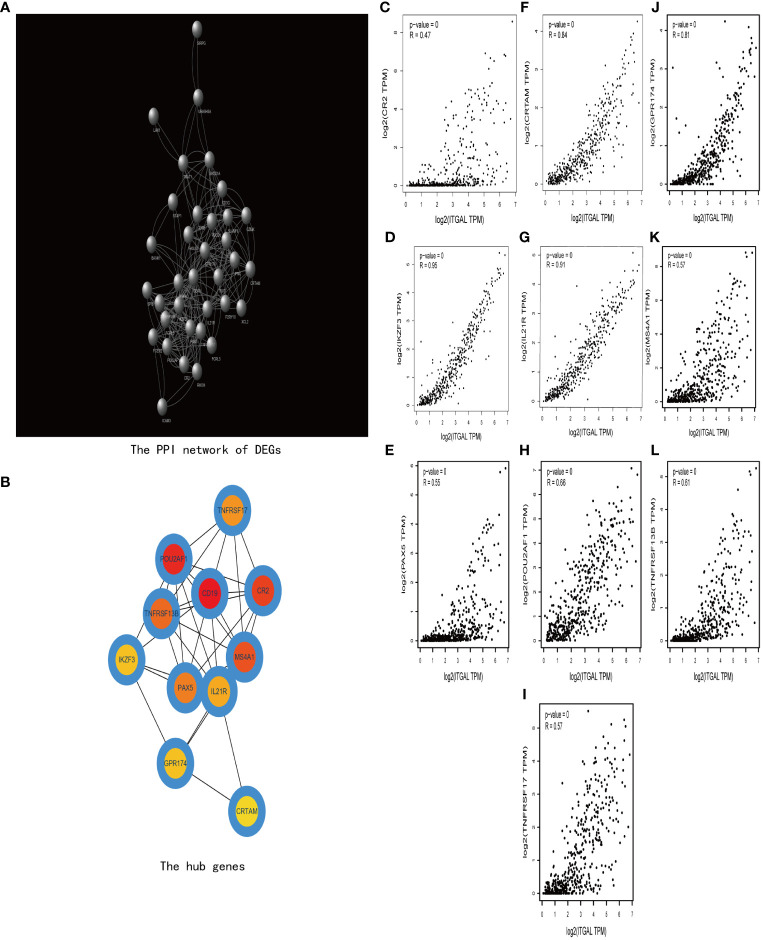
**(A)** The PPI network of DEGs. **(B)** The hub genes of DEGs. **(C–I)** The correlation between ITGAL and the hub genes, including CRTAM, IKZF3, GPR174, IL-21, and TNFRSF13B.

Then we construct a PPI network using TOP 200 DEGs to explore the related functions of differential genes (e.g., **Figure 7A**). Then, using the cytoHubba plug-in (30), we obtained 10 hub genes (e.g., **Figure 7B**). We used GEPIA to explore the relationship between ITGAL and these hub genes in melanoma. We found that CRTAM, IKZF3, GPR174, IL-21, and TNFRSF13B were significantly positively correlated with the expression of ITGAL (e.g., **Figures 7C–N**).

## 4 Discussion

In this study, we explored the expression levels and clinical features of ITGAL in melanoma by systematically analyzing data from public databases and clinically collected samples. Our study revealed that there was a relationship between high expression of ITGAL and a favorable prognosis. Furthermore, our results also showed that different immune cells and immune checkpoints were associated with higher expression of ITGAL. Thus, our study provides a novel perspective and evidence for understanding the critical function of ITGAL in melanoma, which may be an immune infiltration-related prognostic indicator.

We found that ITGAL was aberrantly expressed in different tumor tissues through databases, including GEPIA, Timer, TCGA, and TNMplotter. In particular, the results showed that ITGAL was highly expressed in melanoma. Subsequently, comparing the normal cells and tissue samples, we validated that ITGAL was highly expressed in melanoma samples using qPCR, WB, and mIHC. These results suggest that the expression of the ITGAL may serve as a potential diagnostic indicator for SKCM.

Next, we performed functional enrichment analysis of DEGs between high and low ITGAL, and the results showed that there were obvious correlations between DEGs and cytokine–cytokine receptor interaction, T-cell receptor signaling pathway, B-cell receptor signaling pathway, and hematopoietic cell lineage. It also implicates cytokine–cytokine activity, and the T-cell receptor signaling pathway plays an important role in the progression of ITGAL-mediated melanoma. We performed a correlation analysis on hub genes by using the GEPIA database and identified that the expression of ITGAL was positively correlated with cytokines, including CRTAM, IKZF3, GPR174TL6, IL-21, and TNFRSF13B.

Among these hub genes, class I-restricted T-cell-associated molecule (CRTAM) is a marker of CD4 CTL (31), as well as an early activation marker of NK and CD8+ T cells. CRTAM binds to its ligand, cell adhesion molecule-1 (32, 33). IKAROS Family Zinc Finger 3 (IKZF3) is a transcription factor that plays an important role in the regulation of B-lymphocyte activation, proliferation, and differentiation (34). G protein-coupled receptor 174 (GPR174), which regulate diverse aspects of T-cell activity and effector function, also plays an important role in the suppression of T-cell proliferation (35). IL-21 is a cytokine mainly produced by CD4+ T cells and natural killer T (NKT) cells. IL-21/IL-21 receptor (IL-21R) signaling is important for the proliferation and differentiation of T cells, B cells, natural killer (NK) cells, macrophages, and dendritic cells (DCS) (36, 37). TNFRSF13B is a lymphocyte-specific member of the tumor necrosis factor (TNF) receptor superfamily. This protein induces the activation of the transcription factors NFAT, AP1, and NF-kappa-B and plays a key role in humoral immunity by interacting with TNF ligands (38).

Cytokines are potent regulators of multiple cellular functions and activities, especially in the immune system (25). A review of the literature indicates that elevated expression of these cytokines, which are positively correlated with ITGAL expression, is all involved in promoting tumor cell survival, stemness, and proliferation (26, 27). For example, the tumor necrosis factor (TNF) superfamily can activate signaling pathways that regulate cell survival, death, and differentiation (39).

In the immune infiltration analysis, we found that the expression of ITGAL was significantly associated with multiple immune cells. Including CD4+ T cells, CD8+ T cells, Treg cells, B cells, neutrophils, Tam, DCS, NK cells, and monocytes. CD4+ T cells recognize peptides (about 13–17aa long) bound to the groove of MHC class II molecules (40) ^and^ participate in signal transduction by the T-cell antigen receptor (TCR) (41). In melanoma, CD4+ T cells can also activate CD8+ T cells to differentiate into cytotoxic T lymphocytes (CTLs), while maintaining and boosting the antitumor responses of CTLs (42, 43).

Our results showed that high expression of ITGAL was significantly positively correlated with CD4+ T cells and CD4+ T cells in melanoma. The high expression of ITGAL was involved in the T-cell receptor signaling pathway. Further exploration revealed that ITGAL (CD11a) is a cell surface molecule that forms the LFA-1 complex by binding to the CD18 subunit. LFA-1 is an important adhesion molecule expressed on immune cells that can bind to its ligand, intercellular adhesion molecule-1 (ICAM-1). The LFA-1/ICAM-1 complex plays a role in immune T-cell adhesion, migration, and activation by increasing the production of IL-2 (44, 45), exhibiting anti-inflammatory and anti-tumor effects. ITGAL can promote an increase in the ratio of CD4 and CD8 T cells, improving the survival rate of patients with metastatic melanoma, which may explain why high expression of ITGAL is associated with a better prognosis.

Furthermore, the LFA-1/ICAM-1 complex has been found to be associated with the growth and metastasis of various cancers (46). In particular, the interaction between LFA-1/ICAM-1 and melanoma cells may activate signaling pathways, enhancing the invasive and metastatic abilities of melanoma cells. During the co-culture of melanoma and endothelial cells, Deshayes et al. (44) demonstrated that ICAM-1 and LFA-1 induction allowed melanoma cells to penetrate the endothelial layer *in vitro*, thus enhancing the migration ability of tumor cells. Our mIHC analysis of multiple clinical tissue samples further demonstrated the association between ITGAL and melanoma invasion, but the specific mechanisms still require further exploration through animal experiments.

Immune checkpoint inhibitors (ICIs) are now the mainstay of treatment for melanoma (47). PD1/PDL1 checkpoint blockade therapy is part of the standard therapy for melanoma (48), but because of immune tolerance, it has resulted in poor clinical outcomes in some patients treated with PD-1 pathway blockade (49). Our results show that increased expression of ITGAL is associated not only with PD1 and CTLA4, but also with other potential melanoma checkpoints (LAG-3, Tim-3, and TIGIT).

However, our study has some limitations. First, because most of the results are based on online platform databases, which would be discrepant, and second, further experiments are required to validate immune-related molecular mechanisms underlying the function of ITGAL in melanoma, which will be a future study.

## Data availability statement

The raw data supporting the conclusions of this article will be made available by the authors, without undue reservation.

## Ethics statement

The studies involving human participants were reviewed and approved by Affiliated Hospital of Yangzhou University Ethics Committee, Affiliated Hospital of Yangzhou University. The patients/participants provided their written informed consent to participate in this study.

## Author contributions

TD conceived of the presented idea and finished the work. CW provided technical guidance in the biological experiment. CG helped with the clinical ethics application. QZ and JG provided writing and editing guidance. All authors contributed to the article and approved the submitted version.
